# Editorial: Humanized Mouse Models to Study Immune Responses to Human Infectious Organisms

**DOI:** 10.3389/fimmu.2022.860029

**Published:** 2022-03-10

**Authors:** Melissa Xin Yu Wong, Qingfeng Chen

**Affiliations:** ^1^ Cardiovascular and Metabolic Disorders, Duke-NUS Graduate Medical School, Singapore, Singapore; ^2^ Institute of Molecular and Cell Biology, Agency for Science, Technology and Research (ASTAR), Singapore, Singapore; ^3^ Department of Microbiology and Immunology, Yong Loo Lin School of Medicine, National University of Singapore, Singapore, Singapore

**Keywords:** humanized mouse models, infectious diseases, human specificity, inflammation, liver humanization

This Research Topic is a collection of 9 articles covering the latest development in humanized mice technologies pertaining to immune responses towards infectious organisms. Ranging from bacterial infections that rapidly rot tissue and bone, to chronic viral infections with the ability to evade eradication for decades - each organism distinctly interacts and manipulates the human immune response depending on the environment. This begets the need for a model that can faithfully capture the human immune signature and the host-pathogen interaction. This Topic discusses the advances in 2 types of humanized mice: liver-humanized mice, and mice reconstituted with components of the human immune system.

Vector-borne diseases account for close to 20% of all infectious diseases worldwide. There were an estimated 241 million cases of malaria worldwide, resulting in hundreds of thousands of deaths, especially in children under 5. Modelling the life cycle of malaria parasites has been challenging as human red blood cells (hRBCs) are rapidly eliminated *in vivo*. Existing immunodeficient models require daily transfusions to replenish hRBCs, drastically limiting the scope of preclinical testing for therapeutics. Yamaguchi et al. present a novel NOG sub-strain harboring a murine C3 mutation preventing rejection of hRBCs *in vivo*. This allows hRBCs to be retained in humanized mice up to a remarkable 30 days, thus expanding the scenarios that can be studied *in vivo*, such as vaccinations and co-infections.

Zika virus (ZIKV) is another vector-borne virus known for its devastating neurological and obstetric complications. The search for a ZIKV vaccine remains a highly active field of research. Tarbe et al. demonstrate that memory CD8 T cells generated from Japanese Encephalitis Virus (JEV) immunization are cross-reactive against ZIKV infection in HLA-transgenic mice. Live attenuated JEV vaccinations, along with other vector-borne disease vaccines, have been well studied and used clinically. This discovery is an important step in understanding whether cross-protection among these vaccines exist. Perhaps the solution to a longstanding global health problem could lie in evidence-based repurposing of existing resources, making it not too far out of reach.

Hepatitis B virus (HBV) is notoriously difficult to model *in vivo* due to its strong human hepatotropism. Lai et al. discuss how human liver chimeric mice, among a small handful of animals that successfully harbour HBV infection, remain at the forefront of *in vivo* modelling due to their cost efficiency and availability. With current technologies optimized to produce liver humanization exceeding 70%, researchers are now able to study the life cycle of HBV in a large number of animals. Furthermore, headway has been made into dual humanization models, which provides a powerful pre-clinical platform for recapitulating parts of the human immune response to HBV and therapeutic effects.

Despite the discovery of antiretroviral therapy, Human Immunodeficiency Virus-1 (HIV-1) has evaded eradication for decades and remains a lifelong disease. Gillgrass et al. succinctly summarise the plethora of humanized mouse models that have been developed to understand the pathogenesis of HIV-1, and how it sustains its reservoir in humans. Critically, the article also discusses the use of humice in dissecting the many confounding factors in patient prognosis, including co-infections, the efficacy of HIV vaccines, and immune system interaction with the endogenous microbiome. Gillgrass’ review dovetails nicely with Abeynaike and Paust‘s article on novel HIV-1 therapies studied in humanized mice, featuring gene-editing strategies like CRISPR/Cas9, as well as immune-based therapies such as Chimeric Antigen Receptor (CAR) T cell immunotherapy.

The strength of current humanized mice technology infectious lies in its ability to initiate innate immune responses and antigen-specific T cell responses. However, the full spectrum of humoral response to diseases is limited by the lack of mature B cells and hence antibody class switching. To address this, Cheng et al. presents a proof-of-concept study where TLR9 agonist CpG-B, in combination with co-stimulatory molecule CD40 targeting, triggers a more efficient transition human B cell maturation in humanized mice. This promotes isotype switching from IgM to IgG, the latter playing an essential role in measuring the human response against infectious diseases, thus adding a new dimension to disease modelling capabilities.

Improved B cell maturation in humanized mice also spells good news for researchers studying lymphoproliferative infectious diseases such as Epstein Barr virus (EBV). The two EBV strains vary in their tissue tropism, thus influencing their oncogenic properties. Schuchmachers and Münz review how humanized mice have allowed researchers to tease out the functional significance of viral structures, and how they interact with host genomics and other co-infections to produce malignancy.

Finally, humanized mice have lent great insight into the pathogen-host interaction in tissue infections. Tackling the study of polymicrobial synergy and dysbiosis of the subgingivial microbiota is no easy feat. Rojas et al. discuss at great length the new ways researchers have inoculated Their article covers the immunotherapies that have sprung forth from such studies. Muthukrishnan et al. also present original research on a model recapitulating S. aureus pathogenesis during osteomyelitis, bringing fresh hope for non-antibiotic interventions to combat implant-associated osteomyelitis, as well as to curb rampant multi-drug resistance.

Humanized mice encompass a powerful platform for interrogating the complexity of infectious disease interactions using an *in vivo* biological system. With infectious diseases evolving so rapidly in an interconnected world, technology must swiftly advance to suit the complexity of real-world issues. Constant sharing of knowledge and a strong network will be crucial in maintaining replicability between models and definitively drive progress in an exciting and dynamic field.

## Author Contributions

All authors contributed to the article and approved the submitted version.

## Funding

MW is supported by Duke-NUS MD. Ph.D. program. QC is funded by the National Research Foundation Singapore Fellowship (NRF-NRFF2017-03, NRF-ISF joint grant (NRF2019-NRF-ISF003-3127) and National Medical Research Council- OF-LCG (OFLCG19May-0038).

## Conflict of Interest

QC is the scientific founder of two biotech companies.

The remaining author declare that the research was conducted in the absence of any commercial or financial relationships that could be construed as a potential conflict of interest.

## Publisher’s Note

All claims expressed in this article are solely those of the authors and do not necessarily represent those of their affiliated organizations, or those of the publisher, the editors and the reviewers. Any product that may be evaluated in this article, or claim that may be made by its manufacturer, is not guaranteed or endorsed by the publisher.

